# Acute atomoxetine treatment of younger and older children with ADHD: A meta-analysis of tolerability and efficacy

**DOI:** 10.1186/1753-2000-2-25

**Published:** 2008-09-15

**Authors:** Christopher J Kratochvil, Denái R Milton, Brigette S Vaughan, Laurence L Greenhill

**Affiliations:** 1 University of Nebraska Medical Center, 985581 Nebraska Medical Center, Omaha, NE 68198-5581, USA; 2 Eli Lilly and Company, Lilly Corporate Center, Indianapolis, IN 46285, USA; 3 New York State Psychiatric Institute, 1051 Riverside Drive, New York, NY 10032, USA

## Abstract

**Background:**

Atomoxetine is FDA-approved as a treatment of attention-deficit/hyperactivity disorder (ADHD) in patients aged 6 years to adult. Among pediatric clinical trials of atomoxetine to date, six with a randomized, double-blind, placebo-controlled design were used in this meta-analysis. The purpose of this article is to describe and compare the treatment response and tolerability of atomoxetine between younger children (6–7 years) and older children (8–12 years) with ADHD, as reported in these six acute treatment trials.

**Methods:**

Data from six clinical trials of 6–9 weeks duration were pooled, yielding 280 subjects, ages 6–7 years, and 860 subjects, ages 8–12 years with Diagnostic and Statistical Manual of Mental Disorders, Fourth Edition (DSM-IV)-diagnosed ADHD. Efficacy was analyzed using the ADHD Rating Scale-IV (ADHD-RS), Conners' Parent Rating Scale-revised (CPRS-R:S), and the Clinical Global Impression of ADHD Severity (CGI-ADHD-S).

**Results:**

Atomoxetine was superior to placebo in both age categories for mean (SD) change in ADHD-RS total, total T, and subscale scores; 3 CPRS-R:S subscales; and CGI-ADHD-S from baseline. Although there were no significant treatment differentials between the age groups for these efficacy measures, the age groups themselves, regardless of treatment, were significantly different for ADHD-RS total (younger: ATX = -14.2 [13.8], PBO = -4.6 [10.4]; older: ATX = -15.4 [13.2], PBO = -7.3 [12.0]; p = .001), total T (younger: ATX = -15.2 [14.8], PBO = -4.9 [11.2]; older: ATX = -16.4 [14.6], PBO = -7.9 [13.1]; p = .003), and subscale scores (Inattentive: younger: ATX = -7.2 [7.5], PBO = -2.4 [5.7]; older: ATX = -8.0 [7.4], PBO = -3.9 [6.7]; p = .043; Hyperactive/Impulsive: younger: ATX = -7.0 [7.2], PBO = -2.1 [5.4]; older: ATX = -7.3 [7.0], PBO = -3.4 [6.3]; p < .001), as well as the CGI-ADHD-S score (younger: ATX = -1.2 [1.3], PBO = -0.5 [0.9]; older: ATX = -1.4 [1.3], PBO = -0.7 [1.1]; p = .010). Although few subjects discontinued from either age group due to adverse events, a significant treatment-by-age-group interaction was observed for abdominal pain (younger: ATX = 19%, PBO = 6%; older: ATX = 15%, PBO = 13%; p = .044), vomiting (younger: ATX = 14%, PBO = 2%; older: ATX = 9%, PBO = 6%; p = .053), cough (younger: ATX = 10%, PBO = 6%; older: ATX = 3%, PBO = 9%; p = .007), and pyrexia (younger: ATX = 5%, PBO = 2%; older: ATX = 3%, PBO = 5%; p = .058).

**Conclusion:**

Atomoxetine is an effective and generally well-tolerated treatment of ADHD in both younger and older children as assessed by three recognized measures of symptoms in six controlled clinical trials.

**Trial Registration:**

Not Applicable.

## Background

Attention-deficit/hyperactivity disorder (ADHD) is characterized by developmentally inappropriate levels of inattention, hyperactivity, and impulsivity [1]. In order to make a diagnosis of ADHD, an onset of impairing symptoms is required prior to 7 years of age [1]. Symptoms of ADHD are often present as young as 3 years of age, with epidemiological data suggesting that approximately 2% of children between the ages of 3–5 years meet the Diagnostic and Statistical Manual of Mental Disorders, Fourth Edition (DSM-IV) diagnostic criteria for ADHD [2].

The preschool and early years of school are times of rapid growth and development in children. Failing to identify and treat ADHD early can allow impaired functioning to persist in multiple domains throughout critical periods of development. Preschool children with ADHD are at greater risk for behavioral, academic, social, and family difficulties relative to their unaffected counterparts. In a study of 94 preschool children, those with ADHD had already demonstrated a difference in behavioral ratings that was two standard deviations greater than the control group [3]. By the time children with ADHD enter school, they are likely to be behind their peers without ADHD in basic math concepts, pre-reading skills, and fine motor abilities [4-6].

Even with growing awareness of the potential impairments of ADHD in early childhood, limited data exist regarding its treatment in young children. For example, despite being one of the largest and most influential studies of pediatric psychopharmacology to date, the Multimodal Treatment Study of Children with ADHD (MTA) [7] did not include children under the age of 7 years. The Preschool ADHD Treatment Study (PATS), however, recently assessed the use of methylphenidate (MPH) in preschool children with ADHD [8] in an 8-phase, 70-week, multi-center, randomized efficacy trial. A total of 165 children aged 3.5 to 5.5 years were randomized to treatment with TID MPH. Significant decreases in ADHD symptoms were found at MPH doses of 2.5, 5.0, and 7.5 mg TID (p < .01, p < .001, and p < .001, respectively) when compared with placebo. Effect sizes (0.4–0.8), however, were smaller than those for school-aged children [8]. Relative to the school-aged children in the MTA Study, the preschool group in the PATS study demonstrated a higher rate of emotional adverse effects, including crabbiness, irritability, and proneness to crying [9].

Atomoxetine (ATX), a selective noradrenergic reuptake inhibitor, is a non-stimulant medication approved for the treatment of ADHD in patients 6 years of age through adulthood. No known controlled studies of non-stimulant medications for young children with ADHD have been completed to date, although a small open label 8-week study of ATX in 5- and 6-year old children with ADHD was recently conducted by Kratochvil, et al. [10]. In this study, 22 children were treated with flexibly dosed ATX titrated to a maximum of 1.8 mg/kg/day, with a mean final daily dose of 1.25 mg/kg/day. A significant decrease was observed on the ADHD-IV-RS-Parent total and subscale scores (p < 0.001). Mood lability, described as "angry/hostile", "brittle mood", "emotionally labile", "fussy", "mopey", "rapid mood swings", "tearful" and "irritability", was reported in over half of the subjects (n = 12, 54.5%), and 50% of subjects reported decreased appetite. There were no discontinuations due to adverse events. Vital sign changes were mild and not clinically significant; however, a mean 1.04 kg weight loss was observed for the group (p < 0.001). A larger randomized placebo-controlled trial of ATX in 5- and 6-year olds is underway, and will provide important information on the use of this non-stimulant medication in a younger population.

Allen and Michelson [11] described the extensive process related to the development and FDA approval of ATX as a treatment for ADHD in children. To date, over 4,000 children have participated in Eli Lilly sponsored clinical trials of ATX, including 7 pediatric trials, of which 6 were a randomized, double-blind, placebo-controlled design [12-14]. This large pool of data allows for the evaluation of subpopulations and their variations in treatment response and tolerability. For example, an earlier analysis by Wilens et al. [15] compared children ages 6–11 to adolescents 12–17, demonstrating no statistically significant differences in the overall effects on ADHD symptoms, response rates, or time to response between these age groups. This report will describe and compare the safety, tolerability and efficacy of ATX for the treatment of ADHD in young children, 6–7 years of age, compared with older children, 8–12 years of age.

## Methods

### Subjects

This report is based upon a meta-analysis of 6 randomized, double-blind, placebo-controlled studies of ATX that were conducted in the United States [12] between 1998 and 2004[13,14]. Subjects were 6–16 years of age, although this analysis will focus only on children 6–12 years of age.

### Inclusion and Exclusion Criteria

Subjects were assessed using the Kiddie Schedule for Affective Disorders and Schizophrenia for School-aged Children, Present and Lifetime Versions (KSADS-PL) [16], a semi-structured interview for psychiatric disorders. All subjects were required to meet the DSM-IV [1] diagnostic criteria for ADHD on the KSADS-PL, which was confirmed as the primary diagnosis by clinical assessment.

Although learning disabilities were not exclusionary, subjects were required to be of normal intelligence (IQ ≥ 80) as assessed by one of the following means: four subtests (e.g. Block Design, Picture Arrangement, Similarities, and Vocabulary) of the Wechsler Intelligence Scale for Children-3rd Edition (WISC-III) [17], the full WISC-III, or the general assessment of the physician investigator (studies HFBK, HFBD and LYAT) [13,14]. In three studies (studies LYBG, LYBI and LYCC) [12,18,19], the IQ requirement was ≥70 based on the investigator's assessment of the child. Potential subjects with any serious medical illness, comorbid psychosis or bipolar disorder, history of a seizure disorder, comorbid condition requiring use of excluded concomitant medications, or ongoing use of psychoactive medications other than the study drug, were excluded.

For each subject, a parent or guardian provided written informed consent to participate and the child provided written assent, prior to receiving any study treatment or undergoing any study procedure. These studies met all federal and local regulatory requirements and were conducted in accordance with the ethical standards of each investigative site's institutional review board and the Helsinki Declaration of 1975, as revised in 2000 [20].

### Measures

The primary outcome measure for all 6 studies was the ADHD-RS [21], an investigator-administered and scored instrument that includes the 18 DSM-IV symptom criteria for ADHD. Each item was rated 0–3 by the investigator during a semi-structured interview with the parent or primary caregiver. Subjects were required to have a total score or subscale score that was ≥1.5 standard deviations above age and gender norms, depending on their diagnostic subtype (e.g., total score for combined, or subscale score for primarily inattentive or primarily hyperactive/impulsive). Other measures included the CPRS-R:S [22], which contains subscales for oppositional behavior, hyperactivity and cognition, as well as an ADHD Index, and the CGI-ADHD-S [23]. The ADHD-RS and the CGI-ADHD-S were administered at each visit, while the CPRS-R:S was administered at baseline and again at the final acute treatment visit in all studies.

### Study Design

In 3 studies [12,13,19], the subjects were randomly assigned to receive either once-daily ATX or placebo (PBO) for 6 to 8 weeks. In 2 of the studies [12,19] subjects assigned to ATX received 0.8 mg/kg/day in the morning for 3 days, after which the dose was increased to 1.2 mg/kg/day. These subjects were maintained on an "optimal" dose for 2 to 8 weeks. In the third study [13], subjects assigned to receive ATX had treatment initiated at a dose of 0.5 mg/kg/day for 3 days, after which the dose was increased to 0.75 mg/kg/day for the remainder of the first week. The daily dose was increased to 1.0 mg/kg/day after 7 days of treatment and maintained on an optimal dose for 4 to 6 weeks. In all 3 trials, subjects with significant residual symptomatology (defined as having a CGI-ADHD score ≥3) after 3 to 4 weeks of ATX treatment and without safety or tolerability contraindications could have their dose increased at physician discretion to a maximum of 1.4/1.8 mg/kg/day.

In 2 of the studies [14], design was identical, PBO or double-blinded ATX was dosed twice daily for 9 weeks duration. The titration was flexible based on therapeutic response and tolerability. Atomoxetine doses ranged from 5 to 45 mg BID, with a maximum total daily dose of 90 mg/day permitted, and a maximum weight-adjusted daily dose of 2.0 mg/kg/day. Final visit mean and median doses of ATX in these combined studies were 1.5 and 1.7 mg/kg/day, respectively.

In the final study (study LYBI) [18], subjects were randomized to receive one of three treatments, ATX, PBO, or OROS MPH, for 6 weeks during the acute treatment phase of the trial (Note: Only data from the ATX and PBO treatment groups are included in the present meta-analysis). Subjects assigned to ATX initiated treatment at a dose of 0.8 mg/kg/day divided BID for 4 days, which was then increased to 1.2 mg/kg/day. Similar to the once-daily trials, subjects with significant residual symptomatology (defined as having CGI-ADHD-S score ≥3) after 3 weeks of ATX treatment and without safety or tolerability contraindications could have their dose increased to a maximum of 1.8 mg/kg/day.

### Data Analysis

Age was dichotomized into two categories: 6–7 years; and 8–12 years. Only subjects aged 6 to 12 years were included in this analysis, since 12 was the maximum age for inclusion in all but two of the studies (in studies LYBI and LYAT the maximum age was 16) [13,18]. Patient demographics and baseline characteristics were summarized using descriptive statistics. Change from baseline to endpoint, using a last-observation-carried-forward (LOCF) approach, was computed for all subjects with baseline and at least one post-baseline measurement. For continuous efficacy variables, treatment difference within each age group was assessed by analysis of covariance (ANCOVA) with terms for baseline, protocol, and treatment. Using the ANCOVA model, effect size (ES) was computed by subtracting the least-squares (LS) means for the PBO group from the LS means from the ATX group and dividing by the square root of the mean-squared error. In addition, consistency of treatment effect between age groups for continuous measures was assessed using an ANCOVA model with terms for baseline, protocol, treatment, age group, and treatment-by-age-group interaction.

Response was defined in two different ways in this meta-analysis: 1) ≥25% reduction from baseline in ADHD-RS total score and 2) ADHD-RS total T-scores of < 65. In addition, remission was defined as ADHD-RS total T-scores of < 60 at endpoint. For each response and remission rate, treatment differences within each age group were determined using a Fisher's exact test, while the Breslow-Day test compared odds ratios between the age categories for consistency of treatment effect across the groups. All randomly assigned subjects who took at least one dose of study drug were included in the safety analysis. Treatment-emergent adverse events (AEs) were defined as events that had newly occurred or had worsened after initiating protocol treatment. Treatment-emergent AEs were analyzed similarly to that of response rate. Although height and weight were collected, only weight data are presented due to the short duration of the studies. Pulse and blood pressure were reported for 3 of the 4 studies, but methods of collection were varied (e.g. standing and supine in one study, seated in the other three studies). For change in weight, vital signs, corrected QT interval, and laboratory parameters, treatment difference within each age category was assessed using an ANOVA model with a treatment term. Consistency of treatment effect across age groups was assessed using an ANOVA model with terms for treatment, age group, and treatment-by-age-group interaction. Since laboratory data tended not to meet normality assumptions, ranked data were used instead of raw data in these ANOVA models for all laboratory measures.

All tests were performed using a 2-sided test at a 0.05 significance level, with the exception of the treatment-by-age-group interaction tests, which were performed at a 0.10 significance level. All statistical analyses were performed using SAS software, version 8.2 [24].

## Results

### Demographics

Demographic characteristics for patients by each age category are presented in Table 1. There were 1,140 subjects in the pooled analysis, of which 280 (25%) were 6–7 years of age (ATX, n = 184; PBO, n = 96) while 860 patients (75%) were 8–12 years of age (ATX, n = 544; PBO, n = 316). Seventy-four percent of the subjects were male, and 71% were Caucasian. The mean ages were 7.2 years in the 6- to 7-year-old group and 10.2 years for the 8- to 12-year-old group. Seventy-three percent of all subjects met criteria for ADHD, combined subtype; 24% were classified as inattentive subtype and 2% were classified as hyperactive/impulsive subtype. There were no statistically significant demographic differences found between ATX and PBO treatment groups within each age group.

**Table 1:** Summary of Demographic Characteristics

	6- and 7-Year Olds			8- to 12-Year Olds		
	
	ATX	PBO		ATX	PBO	
Subject Characteristics	N = 184	N = 96	p^a^	N = 544	N = 316	p^a^
Gender, n (%)						
Female	54 (29.3)	22 (22.9)	.262	137 (25.2)	78 (24.7)	.935
Male	130 (70.7)	74 (77.1)		407 (74.8)	238 (75.3)	
Age (years), mean (SD)	7.2 (0.6)	7.1 (0.5)	. 273^b^	10.2 (1.4)	10.2 (1.4)	.606^b^
Origin, n (%)						
African descent	16 (8.7)	15 (15.6)	.179	81 (14.9)	40 (12.7)	.442
Caucasian	137 (74.5)	64 (66.7)		374 (68.8)	234 (74.1)	
Hispanic	22 (12.0)	9 (9.4)		59 (10.8)	27 (8.5)	
Other	9 (4.9)	8 (8.3)		30 (5.5)	15 (4.7)	
Prior stimulant treatment, n (%)						
No	133 (72.3)	68 (70.8)	.889	235 (43.3)	141 (44.6)	.722
Yes	51 (27.7)	28 (29.2)		308 (56.7)	175 (55.4)	
ADHD Subtype, n (%)						
Hyperactive/impulsive	10 (5.4)	2 (2.1)	.395	10 (1.8)	5 (1.6)	.828
Inattentive	26 (14.1)	12 (12.5)		148 (27.2)	92 (29.1)	
Combined	148 (80.4)	82 (85.4)		386 (71.0)	219 (69.3)	
ADHD Severity, mean (SD)						
Baseline ADHD Total T score	83.4 (9.5)	83.2 (8.7)	.862	81.7 (11.6)	81.2 (11.2)	.512
Baseline CGI-ADHD-S score	5.0 (0.8)	5.0 (0.7)	.743	4.9 (0.8)	4.9 (0.8)	.909

Abbreviations: ADHD = attention-deficit/hyperactivity disorder; ATX = atomoxetine; PBO = placebo; SD = standard deviation

^a ^p values were for comparing atomoxetine and placebo using a Fisher's exact test.

^b ^p values were for comparing atomoxetine and placebo using an ANOVA.

### Baseline Characteristics

ADHD symptom severity was similar at baseline for both treatment conditions within each age group, as measured by the ADHD-RS total T score and the CGI-ADHD-S score. However, between age groups, younger subjects (6–7 years) experienced more severe ADHD symptoms at baseline compared with the older subjects (8–12 years). Mean ADHD-RS total T scores were at least 3 standard deviations above normal in each group. A higher percentage of children in the older age group met criteria for the inattentive subtype compared with those in the younger group. More children aged 8–12 years had previously been treated with a stimulant compared with their younger counterparts. Comorbid conditions were comparable, with 34% of the subjects in both age groups meeting diagnostic criteria for oppositional defiant disorder (ODD).

### Efficacy

Table 2 summarizes the change from baseline to endpoint for ADHD-RS total score, subscale scores, and total T-score, CGI-ADHD-S score, and all four CPRS-R:S subscale scores. With the exception of the CPRS-R:S Oppositional subscale, ATX-treated subjects in both the younger and older age groups demonstrated significant improvement compared with those treated with PBO on all efficacy measures: ADHD-RS total score: younger ES = .77, older ES = .65; total T-score: younger ES = .75, older ES = .63; Inattentive subscale: younger ES = .71, older ES = .59; Hyperactive/Impulsive subscale: younger ES = .76, older ES = .62; CGI-ADHD-S score: younger ES = .62, older ES = .59; CPRS-R:S ADHD Index: younger ES = .50, older ES = .74; CPRS-R:S Cognition: younger ES = .41, older ES = .69; CPRS-R:S Hyperactive: younger ES = .56, older ES = .72). In addition, only the CPRS-R:S Oppositional subscale had a statistically significant treatment-by-age-group interaction. However, significant age group differences were observed for ADHD-RS total and subscale scores and CGI-ADHD-S score, where older children (irrespective of whether they were treated with ATX or PBO) improved significantly more than their younger counterparts.

**Table 2:** Summary of Efficacy Measures – Change from Baseline to Endpoint

			Baseline			Change			Vs. Placebo	Subgroup	Treatment by Subgroup
			
Measure	Subgroup (yrs)	Tx	N	Mean	SD	Mean	SD	ES	p Value^a^	p Value^b^	p Value^b^
ADHD-RS Total	6–7	ATX	176	42.8	7.9	-14.2	13.8	.77	< .001	.001	.316
		PBO	91	43.2	6.6	-4.6	10.4				
	8–12	ATX	520	40.4	8.7	-15.4	13.2	.65	< .001		
		PBO	303	40.0	8.2	-7.3	12.0				
ADHD-RS Total T-Score	6–7	ATX	176	83.3	9.6	-15.2	14.8	.75	< .001	.003	.346
		PBO	91	83.1	8.5	-4.9	11.2				
	8–12	ATX	520	81.5	11.4	-16.4	14.6	.63	< .001		
		PBO	303	81.1	10.9	-7.9	13.1				
ADHD-RS Inattentive	6–7	ATX	176	21.9	3.8	-7.2	7.5	.71	< .001	.043	.439
		PBO	91	22.1	3.7	-2.4	5.7				
	8–12	ATX	520	22.4	3.7	-8.0	7.4	.59	< .001		
		PBO	303	22.3	3.9	-3.9	6.7				
ADHD-RS Hyper/Impulsive	6–7	ATX	176	20.9	5.6	-7.0	7.2	.76	< .001	< .001	.257
		PBO	91	21.2	4.5	-2.1	5.4				
	8–12	ATX	520	18.0	6.7	-7.3	7.0	.62	< .001		
		PBO	303	17.7	6.3	-3.4	6.3				
CGI-ADHD-S	6–7	ATX	176	5.0	0.8	-1.2	1.3	.62	< .001	.010	.800
		PBO	91	5.0	0.7	-0.5	0.9				
	8–12	ATX	520	4.9	0.8	-1.4	1.3	.59	< .001		
		PBO	304	4.9	0.8	-0.7	1.1				
CPRS-R:S ADHD Index	6–7	ATX	83	27.5	6.0	-7.1	11.2	.50	.009	.723	.422
		PBO	42	28.6	5.0	-3.2	8.4				
	8–12	ATX	290	27.3	6.0	-8.1	8.7	.74	< .001		
		PBO	188	27.1	6.1	-2.1	8.5				
CPRS-R:S Cognitive	6–7	ATX	83	13.7	3.5	-3.6	6.0	.41	.033	.614	.334
		PBO	41	14.0	3.3	-1.6	4.8				
	8–12	ATX	289	13.9	3.8	-4.0	5.1	.69	< .001		
		PBO	188	13.9	3.8	-0.8	5.2				
CPRS-R:S Hyperactive	6–7	ATX	83	12.4	4.3	-3.9	5.7	.56	.004	.095	.753
		PBO	42	13.3	3.2	-1.6	4.9				
	8–12	ATX	290	10.4	5.1	-4.1	4.5	.72	< .001		
		PBO	188	10.1	4.8	-1.1	4.0				
CPRS-R:S Oppositional	6–7	ATX	83	9.4	4.6	-1.9	5.4	.31	.104	.256	.090
		PBO	42	8.3	5.0	0.1	3.8				
	8–12	ATX	290	8.7	4.7	-1.4	4.2	.05	.620		
		PBO	188	8.8	4.6	-1.2	4.1				

Abbreviations: ADHD = attention-deficit/hyperactivity disorder; ADHD-RS = ADHD Rating Scale-IV; ANCOVA = analysis of covariance;

ATX = atomoxetine; CPRS-R:S = Conners' Parent Rating Scale-revised; CGI-ADHD-S = Clinical Global Impression of ADHD Severity scale;

ES = effect size; PBO = placebo; SD = standard deviation.

^a^p values comparing ATX vs. PBO by subgroup are based on an ANCOVA on change from baseline scores with terms for baseline, protocol, and treatment.

^b^p values for subgroup and treatment-by-subgroup interaction are based on an ANCOVA on change from baseline scores with terms for baseline, protocol, treatment, subgroup, and treatment-by-subgroup interaction.

Response rates, defined as ≥25% reduction from baseline in ADHD-RS total score, were significantly different between ATX and PBO treatment groups for children 6–7 years (ATX, 55.7%; PBO, 22.0%; p < .001) and children aged 8–12 years (ATX, 63.5%; PBO, 35.6%; p < .001). No statistically significant differential treatment effects were observed between the age groups (p = .287). Response rates, defined as having endpoint T-scores of < 65, were significantly different between ATX and PBO treatment groups for the 6–7 year olds (ATX, 44.3%; PBO, 16.5%; p < .0001) and the 8–12 year olds (ATX, 51.9%; PBO, 28.4%; p < .0001). The treatment-by-age-group effect was not significant (p = .270). The percentage of subjects experiencing remission at endpoint, as defined by T-score < 60, were significantly different between ATX and PBO for both age groups (6–7 year old ATX, 36.4%, PBO, 8.8%, p < .0001; 8–12 year old ATX, 41.0%, PBO, 19.8%, p < .0001). A significant treatment-by-age effect was seen (p = .0830) in remission rates.

### Safety and Tolerability

Atomoxetine was well tolerated by children in both age groups. The median and mean (standard deviation [SD]) final ATX doses were 1.47 mg/kg/day and 1.39 (0.38) mg/kg/day for younger children, and 1.44 mg/kg/day and 1.37 (0.40) mg/kg/day for older children. The difference in final dose was not statistically significant. Rates of study completion were similar between the two groups (younger children, 76.4%; older children, 78.5%).

Reasons for discontinuation for subjects receiving ATX or PBO did not significantly differ within age groups, with the exception of discontinuation due to lack of efficacy. Patients who received PBO had a significantly higher rate of study discontinuation due to lack of efficacy for both younger (ATX, 1.1%; PBO, 6.3%; p = .021) and older (ATX, 2.8%; PBO, 9.5%; p < .001) children. Conversely, incidence of study discontinuation due to AEs was not significantly different between treatment groups in younger (ATX, 1.1%; PBO, 4.2%; p = .186) versus older (ATX, 3.7%; PBO, 1.6%, p = .093) children. However, a significant differential treatment effect was observed between the age groups (p = .015).

Treatment-emergent AEs reported by at least 5% of patients are presented in Table 3. Younger children taking ATX versus PBO had significantly higher rates of upper abdominal pain, decreased appetite, vomiting, and somnolence. Among older children, there were significantly higher rates of decreased appetite, somnolence, irritability, and fatigue observed for those taking ATX versus PBO. Of these treatment-emergent AEs, upper abdominal pain and vomiting had a significant treatment-by-age-group interaction. The odds ratio (OR) for treatment-emergent upper abdominal pain in younger versus older children was 3.4 and 1.2, respectively (p = 0.044); for vomiting, the OR was 7.4 and 1.4, respectively, for younger versus older children (p = 0.053). Of note, a significant treatment differential was also observed for pyrexia and cough.

**Table 3:** Summary of Treatment-Emergent Adverse Events Reported by at Least 5% of Subjects in Either Age Group

	6- and 7-Year Olds			8- to 12-Year Olds			
					
	ATX	PBO		ATX	PBO		
	N = 183	N = 95		N = 542	N = 316		
					
Event	n (%)	(n) %	p Value^a^	(n) %	(n) %	p Value^a^	p Value^b^
Abdominal pain upper	34 (18.6)	6 (6.3)	.006	83 (15.3)	40 (12.7)	.313	.044
Decreased appetite	30 (16.4)	3 (3.2)	< .001	82 (15.1)	17 (5.4)	< .001	.331
Headache	25 (13.7)	8 (8.4)	.243	90 (16.6)	54 (17.1)	.850	.214
Vomiting	25 (13.7)	2 (2.1)	.001	51 (9.4)	18 (5.7)	.068	.053
Cough	18 (9.8)	6 (6.3)	.375	18 (3.3)	28 (8.9)	< .001	.007
Nausea	13 (7.1)	2 (2.1)	.097	42 (7.7)	20 (6.3)	.496	.186
Fatigue	12 (6.6)	2 (2.1)	.150	39 (7.2)	8 (2.5)	.003	.918
Somnolence	12 (6.6)	1 (1.1)	.040	50 (9.2)	14 (4.4)	.010	.294
Irritability	9 (4.9)	2 (2.1)	.342	33 (6.1)	9 (2.8)	.034	.924
Pyrexia	9 (4.9)	2 (2.1)	.342	15 (2.8)	17 (5.4)	.062	.058
Nasopharyngitis	8 (4.4)	4 (4.2)	1.00	26 (4.8)	24 (7.6)	.098	.442
Upper respiratory tract infection	8 (4.4)	3 (3.2)	.754	16 (3.0)	17 (5.4)	.096	.206
Pharyngolaryngeal pain	2 (1.1)	3 (3.2)	.342	26 (4.8)	29 (9.2)	.014	.687

Abbreviations: ATX = atomoxetine; N, n = number; PBO = placebo.

^a^P values comparing ATX and PBO within each subgroup are based on Fisher's exact test.

^b^P values comparing odds ratios between children and adolescent

Atomoxetine was associated with a statistically significant increase in mean (SD) pulse rate for both younger (ATX, 8.7 [12.7]; PBO, 1.0 [13.7]; p = .001) and older (ATX, 6.8 [11.7]; PBO, 0.6 [11.3]; p < .001) subjects. Similarly, a statistically significant treatment-group difference in systolic blood pressure (ATX, 2.1 [9.8] mmHg; PBO, 0.3 [8.1] mmHg; p = .034) and diastolic blood pressure (ATX, 2.9 [8.2] mmHg; PBO, 0.6 [8.0] mmHg; p = .002) was observed for older children, but not for younger children. There was no significant treatment-by-age-group interaction observed for either pulse rate, systolic or diastolic blood pressure. For mean (SD) weight change from baseline to endpoint, a statistically significant decrease in weight was observed for children taking ATX compared with PBO in both age groups (younger: ATX, -0.5 [1.1] kg; PBO, +0.7 [0.7] kg; older: ATX, -0.6 [1.3] kg; PBO, +1.1 [1.4]; p < .001 for both). In addition, a significant differential treatment effect between the age groups was observed for mean weight change (p = .004). There were no significant differences between the age groups or significant treatment-by-age-group interaction for mean (SD) corrected QT interval (Fridericia's method) (younger: ATX, -1.0 [21.1] msec; PBO, 0.7 [16.3] msec; p = .510; older: ATX, -0.9 [18.1] msec; PBO, -1.1 [17.5] msec; p = .862; interaction p = .485), and no clinically meaningful differences in laboratory measures.

## Discussion

ADHD is a disorder that, by definition, presents at a young age and generally persists for years with continuing treatment often recommended. Limited information, however, exists regarding the safety and efficacy of pharmacotherapy for ADHD in children under the age of 8. The current analysis takes advantage of the growing database from multiple clinical trials of ATX to examine differences in response and tolerability in younger (6–7 years) versus older (8–12 years) children with ADHD. In the absence of trials specifically designed to examine these outcomes in young children treated with ATX, this meta-analysis may be the only available means of systematically assessing the effects of this medication, which is used with increasing frequency in this patient population.

As anticipated, ATX was effective in reducing core symptoms of ADHD in both age groups. There was a statistically significant improvement compared with placebo for both age groups in all but one of the efficacy measures. Combining data from ATX and PBO patients, significant age group differences were observed for ADHD-RS total and subscale scores, as well as the CGI-ADHD-S score, in which older children demonstrated significantly greater improvement compared with younger children.

While generally well-tolerated by both younger and older children with ADHD, ATX treatment in the 6- to 7-year-olds resulted in higher rates of upper abdominal pain, decreased appetite, vomiting, and somnolence compared with PBO, while 8- to 12-year-olds experienced higher rates of decreased appetite, somnolence, irritability, and fatigue. There were also statistically significant increases in pulse and decreases in weight for both younger and older children on ATX treatment compared with PBO. Increases in systolic and diastolic blood pressures in the older children and decreased weight in the younger children, although statistically significant, were not judged as clinically significant. Nonetheless, increases in mean pulse and blood pressures, while not generally clinically significant in this study, are enough to warrant monitoring when utilizing ATX to treat pediatric patients. The laboratory and ECG values revealed no safety concerns, including no evidence of hepatotoxicity. These data support the current guidelines of monitoring children clinically while on ATX treatment without obtaining baseline or ongoing laboratory or ECG evaluations unless a specific clinical presentation is cause for concern (i.e., jaundice, pruritus, or dark urine).

This analysis is limited by the relatively short duration of the 6 studies. Patients were treated for 6 to 9 weeks with either once or twice-daily ATX. Target doses (approximately 1.2–1.5 mg/kg) were achieved over a range of a few days to more than two weeks, depending upon the study. Therefore, maximum benefit from ATX may not have been achieved by all subjects during the treatment period, as total time on target dose may have been insufficient. Procedural variations in timing of doses, titration, duration of the study, and methods of collecting vital signs may have limited the ability to combine and/or interpret the data. Additionally, the omission of a teacher-rated efficacy measure may limit this study's application to a school-based setting.

The long-term safety and efficacy of ATX in young children cannot be determined by the results of this analysis. However, a previous study demonstrated atomoxetine to be effective and generally well-tolerated in 6- and 7-year-olds over a period of up to two years [25].

## Conclusion

The data presented here suggest that the ADHD symptoms of children 6–7 years old improve with ATX treatment, with a more effective overall response compared with that seen in children 8–12 years old. The side effect profile of ATX differed slightly in the younger versus older children, with few study discontinuations from either group due to AEs. These data are important in making clinicians aware that, in general, the response and tolerability of ATX treatment did not vary significantly between these two age groups. However, the potential for these differences must be taken into account when assessing the risk/benefit relationship of the medication and making treatment decisions. Atomoxetine use may warrant additional care and surveillance when treating younger children, about whom we have very limited information. Further research is warranted, particularly to examine atomoxetine long-term safety and efficacy, in the treatment of young children with ADHD.

## List of abbreviations

ADHD: attention-deficit/hyperactivity disorder; ADHD-RS: ADHD Rating Scale-IV; AE: adverse event; ANCOVA: analysis of covariance; ANOVA: analysis of variance; ATX: atomoxetine; CGI-ADHD-S: Clinical Global Impression of ADHD Severity; CPRS-R:S: Conners' Parent Rating Scale-revised; DSM-IV: Diagnostic and Statistical Manual of Mental Disorders, Fourth Edition; ES: effect size; FDA: (U.S.) Food and Drug Administration; IQ: intelligence quotient; KSADS-PL: Kiddie Schedule for Affective Disorders and Schizophrenia for School-aged Children, Present and Lifetime Versions; LOCF: last-observation-carried-forward; LS: least-squares; MPH: methylphenidate; MTA: Multimodal Treatment Study of Children with ADHD; ODD: oppositional defiant disorder; OR: odds ratio; PATS: Preschool ADHD Treatment Study; PBO: placebo; SD: standard deviation; WISC-III: Wechsler Intelligence Scale for Children-3rd Edition.

## Competing interests

Dr. Kratochvil: Honoraria/Consultant, Research Support, and/or Speakers Bureau: Cephalon, Eli Lilly, McNeil, Abbott, Pfizer, Shire, Somerset, AstraZeneca. Ms. Milton is an employee and shareholder of Eli Lilly and Company. Ms. Vaughan has no competing interests to report. Dr. Greenhill: Honoraria/Consultant, Research Support, and/or Speakers Bureau: Celltech, Cephalon, Eli Lilly, Janssen, McNeil, Medeva, Novartis Corporation, Noven, Otsuka, Pfizer, Sanofi, Shire, Solvay, Somerset, Thompson Advanced Therapeutics Communications.

## Authors' contributions

CJK participated in the design of the study, and contributed to the drafting and review of the manuscript. DRM participated in the design of the study, performed the statistical analysis, and contributed to the drafting and review of the manuscript. BSV and LLG contributed to the drafting and review of the manuscript. All authors substantially contributed to the drafting of the manuscript, revising it critically for important intellectual content, and have read and given final approval of the version to be submitted for publication.
